# The Relationship between Serum Trace Elements and Oxidative Stress of Patients with Different Types of Cancer

**DOI:** 10.1155/2021/4846951

**Published:** 2021-07-24

**Authors:** Yu-wei Yang, Chun-mei Dai, Xiao-hong Chen, Jia-fu Feng

**Affiliations:** Department of Clinical Laboratory, Mianyang Central Hospital, School of Medicine, University of Electronic Science and Technology of China, Mianyang 621000, China

## Abstract

**Objective:**

Many studies have identified causal and promotive roles of oxidative stress (OxS) and oxidative damage caused by OxS in the occurrence and progression of cancer. Many biomarkers in the blood circulation of patients may change correspondingly with the development of tumors. This study is aimed at investigating the correlation between OxS and serum trace element (TE) levels of patients with different types of cancer.

**Methods:**

1143 different types of cancer patients and 178 healthy controls from Mar. 2018 to Aug. 2020 in Mianyang Central Hospital were involved in this study. Their levels of OxS parameters (including total oxidant status (TOS), total antioxidant status (TAS), and oxidant stress index (OSI)) and the concentrations of serum TEs (including Cu, Zn, Fe, and Se) were determined.

**Results:**

Compared with healthy controls, all types of cancer patients had higher TOS level (all *P*_adj_ < 0.001) and OSI level (*z* = 6.228 ~ 9.909, all *P*_adj_ < 0.001) and lower TAS level (all *P*_adj_ < 0.001). Compared with healthy controls, the changes of four TE levels in serum were different in different types of cancer patients, among which Cu increased in all groups, but there was no statistical difference in gastric and brain cancer; Se decreased in all groups, but there was no statistical difference in gastric, colorectal, esophageal, and other cancer; Zn was significantly decreased in breast cancer patients (*P*_adj_ < 0.001); there was no statistical difference in the change of Fe in liver, kidney, and other cancer. Spearman correlation showed that the change of Cu concentration was most closely related to the three OxS parameters and was strongly correlated in the observed several types of tumors (*r*_*s*_ > 0.6). Multinomial logistic regression showed that the risks of different tumors are related to the level change of multiple TEs and OxS parameters (OR_TOS_ = 1.19 ~ 2.82, OR_OSI_ = 2.56 ~ 4.70, OR_TAS_ = 0.20 ~ 0.46, OR_Cu_ = 0.73 ~ 1.44, OR_Zn_ = 0.81 ~ 0.91, OR_Fe_ = 0.68 ~ 1.18, and OR_Se_ = 0.22 ~ 0.45, all *P* < 0.006).

**Conclusions:**

The OxS exists in the occurrence and development of cancer, which may be related to the changes of certain trace elements. In order to evaluate OxS correctly, it is necessary to detect TAS and TOS and at the same time, their ratio OSI should be detected. Assessment of markers representing the overall level of OxS and TEs may guarantee improved the monitoring of disease occurrence and development risk in cancer patients.

## 1. Introduction

Chronic infection and/or inflammation has been recognized as an important risk factor for tumorigenesis [1, 2]. It has been shown that active oxygen species generated in inflamed tissues, such as superoxide anion, hydrogen peroxide, and hydroxyl radical, can damage the structure and function of target cells, cause protein denaturation and DNA damage, and promote tumor development [3, 4].

The body prooxidant-antioxidant system is composed of the formation of active oxygen metabolites [i.e., reactive oxygen species (ROS)] and the rate of antioxidant scavenging reactive oxygen metabolites. All aerobic organisms produce free radicals (FR) and reactive oxygen species (ROS) through various oxidase reactions. However, there are also enzymes and nonenzymatic antioxidants to remove FR and ROS generated by the oxidase reaction process [5–7]. Under physiological conditions, the enzymes and nonenzymatic substances in these metabolisms maintain a dynamic equilibrium state of oxidation-antioxidation and commonly participate in a variety of normal physiological functions [8, 9]. However, due to the imbalance between prooxidant and antioxidant components, in many processes, such as inflammation and carcinogenesis, oxidative stress (OxS) is accompanied by an increase in ROS production and/or a decrease in antioxidant levels in target cells and tissues [9–11]. The intracellular oxidative damage is mainly caused by FR and ROS [12–14], which leads to the oxidative damage of protein and DNA [14, 15]. Therefore, this affects intracellular physiological metabolism, activates cancer-related factors, and eventually induces the development and progression of carcinoma [16–18]. In addition, the body constantly consumes antioxidants against oxidative damage, which aggravates the OxS response.

In the previous studies on OxS, most researchers evaluated the change of one or several oxidation or antioxidant substances. However, the substances that maintain the balance of the oxidation-antioxidant system in the body are extremely complex. Some substances are recognized, and there may be unrecognized ones. Therefore, it is impossible to correctly evaluate the subjects' OxS only by observing the changes of certain oxidation and/or antioxidant substances. Total antioxidant status (TAS) represents the total level of antioxidants in the body, and total oxidant status (TOS) represents the total level of oxidants in the body. Oxidant stress index (OSI), which is the ratio of these two, can reflect the imbalance of the ratio of oxidation and antioxidant substances in the dynamic change process, that is, the inconsistency of TAS and TOS changes. Therefore, scholars have used TAS, TOS, and OSI to evaluate the OxS level of cancer patients [19]. As TOS and TAS reflect the overall levels of oxidation and antioxidants in the samples, OSI reflects the balance between them (i.e., oxidation and antioxidation). Therefore, our previous research on thyroid cancer also showed that the patient's OxS status can be better evaluated using these three parameters [20].

Some trace elements (TEs), such as copper (Cu), zinc (Zn), iron (Fe), and selenium (Se), play an important role in many biological processes by activating or inhibiting enzymatic reactions. They can compete with other elements and metalloproteins for binding sites and affect cell membrane permeability or other mechanisms. These TEs can promote lipid peroxidation to generate free radicals and participate in electron transport and initiate free radical chain reactions, which leads to changes in blood composition [21–23]. Se is a component of many antioxidant enzymes, including glutathione peroxidase, superoxide dismutase, and thioredoxin reductase [24]. Most of the biological activities of Se are realized by its binding as a rare amino acid selenocysteine [25]. Similarly, Zn binds to over 2700 enzymes and/or proteins, including hydrolases, oxidoreductase, transferases, isomerases, ligases, and lyases. Zinc protein is equivalent to 10% of human proteome and maintains the composition and structural integrity of many proteins [26]. In mammalian cells, Cu and Fe are the main redox active metals, which catalyze the production of ROS and can oxidize cellular components, such as unsaturated lipid bonds in membrane lipid layer [26]. Se and Zn compete with Cu and/or Fe for negatively charged metals in lipid layers. Therefore, Se and Zn can protect cell membrane from oxidative damage caused by lipid oxidation and avoids OxS [26]. However, once these trace elements in the body lose their balance, they can lead to unsaturated bond reaction in membrane lipids, denaturation of proteins, damage of nucleic acids, and oxidative damage of cells, resulting in OxS. Many studies have shown that these TEs have an effect on the carcinogenic process. Changes in the distribution of these TEs in tissues and serum have been reported in patients with various cancers. However, their exact role in carcinogenesis is still unknown.

In the literature according to our knowledge, no study has previously evaluated the potential association among cancer, trace elements, and oxidative stress therefore provides a comprehensive knowledge to this field by monitoring a variety of highly prevalent cancers. Based on this, the purpose of this study is to explore the relationship between serum TEs and OxS by detecting the levels of four trace elements (including Cu, Fe, Zn, and Se) and total oxidation/antioxidant parameters (including TAs, TOS, and OSI) in 8 most common cancers and 9 common cancer patients.

## 2. Materials and Methods

### 2.1. Subjects

#### 2.1.1. Patients

A total of 1143 cancer patients (679 males and 464 females) were recruited from Mianyang Central Hospital, Sichuan Province, China, from March 1, 2018 to August 31, 2020. These patients included 107 cases of liver cancer, 119 cases of gastric carcinoma, 120 cases of colorectal cancer, 128 cases of breast cancer, 150 cases of lung cancer, 117 cases of esophageal cancer, 115 cases of brain cancer, 156 cases of kidney cancer, and 131 cases of other cancer. The 131 cases of other cancer patients were not singled out for special statistics, due to their slight degree of cases. There were 16 submucous hysteromyomas, 17 cervix cancers, 14 rhinitis cancers, 12 carcinoma of penis, 15 spinal cord cancer (male), 11 ovarian carcinoma, 15 medullary thyroid carcinoma, 17 parotid gland mucoepidermoid carcinoma, and 14 bladder transitional epithelium cancer. The mean age of the cancer patients was 52.4 ± 13.5 years, ranging from 18 to 82 years.

*Inclusion criteria*. (1) Age > 18, (2) carcinoma in situ, (3) without treatment in any way, (4) all diagnoses meet the standards of the National Comprehensive Cancer Network (NCCN), and (5) the patient's survival time after sample collection is more than 6 months to avoid the occurrence of unidentified complications.

*Exclusion criteria*. (1) The tumor has metastasized (to avoid confusion of tumor classification) or recurred; (2) patients had received treatment, such as radiotherapy or chemotherapy; (3) patients took anti-inflammatory drugs or nutritional supplements or antioxidant or vitamin supplements within the past one month; (4) patients had smoking or alcohol abuse one month before enrollment; (5) patients with any other diseases, such as hypoglycemia, diabetes mellitus, gout, thyroid disease, autoimmune disease, liver diseases, primary kidney disease, protein-energy malnutrition, and vitamin A/D deficiency.

#### 2.1.2. Healthy Controls

There were 178 healthy volunteers (95 males and 83 females) as control subjects. Their age, gender, job type, academic occupation, and hobby matched with the total sufferers. The mean age of the healthy controls was 50.3 ± 14.7 years, ranging from 18 to 79 years old. For healthy controls, except for normal liver and kidney function and negative hematuria, they had no symptoms and their clinical sign examination was completely normal. The exclusion criteria of the healthy control group were the same as the patient group.

The Ethics Committee of Mianyang Central Hospital, School of Medicine, University of Electronic Science and Technology of China had approved the protocol in this study. All participants issued written agreements before the experiment.

### 2.2. Sample Collection

After fasting overnight, blood samples of subjects were prepared by collecting venous blood in 5 ml Vacutainer tubes (Becton Dickinson). After 30 min and within 2 h, the blood samples were centrifuged at 3000 rpm for 15 min. The serum was stored in a refrigerator at 2-8°C until analysis within 24 h.

Histopathological examination of tissue samples obtained after operation or organ puncture was performed by standard hematoxylin/eosin staining, and the double blind definitive diagnosis was made by two independent pathologists.

### 2.3. Trace Element Analysis

The concentrations of Cu, Zn, Fe, and Se were measured in the same detection system by NexION® 300 Inductively Coupled Plasma Mass Spectrometry (ICP-MS) (PerkinElmer, USA).

### 2.4. OxS Parameter Measurement

Serum TAS and TOS levels were measured by a LAbOSPECT 008AS automatic biochemical analyzer (Hitachi, Japan). The principle and method are as follows.

#### 2.4.1. TAS

TAS was determined using a modified Erel's TAS colorimetrical method [27]. The assay relied on the ability of antioxidants to promote the reduction of ABTS^+^ to ABTS [2,2′-azino-bis-(3-ethyl-benzothiazoline-6-sulphonate)] in the sample. TAS level was measured based on the change in ABTS^+^. The assay was calibrated with a Trolox standard (6-hydroxy-2,5,7,8-tetramethylchroman-2-carboxylic acid, a water-soluble analog of vitamin E). The results were expressed in mmol/L Trolox equivalent (mmol/L Trolox).

#### 2.4.2. TOS

Serum TOS was measured using Erel's TOS colorimetrical method [28], which relied on the oxidation of ferrous ions to ferric ions in the presence of various oxidative species in an acidic medium. Xylenol orange was used as an indicator reflecting the increase of ferric ion to determine the TOS level. The assay was calibrated with a hydrogen peroxide (H_2_O_2_) standard. The results were expressed in *μ*mol/L H_2_O_2_ equivalent (*μ*mol/L H_2_O_2_).

#### 2.4.3. OSI

The TOS-to-TAS ratio was defined as OxS index (OSI) [29, 30] and calculated as follows: OSI (arbitrary unit) = [(TOS, *μ*mol/LH_2_O_2_)/(TAS, mmol/L Trolox] ÷ 10 [31].

### 2.5. Statistical Analysis

Statistical analysis was performed in MedCalc for Windows, version 18.2 (MedCalc Software, Mariakerke, Belgium) or the Statistical Package for the Social Sciences, version 19.0 (SPSS Inc., Chicago, IL, USA). The measurement results were expressed as the mean plus/minus the standard deviation ($\bar{x}\pm s$) (if normal distribution) or the median and 25th/75th percentiles [*M*(*P*_25_, *P*_75_)] (if nonnormal distribution), and the count results were expressed as cases number and the percentage [*n*(%)]. Differences between two groups were compared using the *t*-test or Mann-Whitney test for the measurement results and the Chi-squared test for the count results. Differences of measurement data among multiple groups were analyzed by the Kruskal-Wallis test, and pairwise comparison was used by post hoc multiple comparison of the Kruskal-Wallis test, and the difference was statistically significant with the adjusted *P* value (*P*_adj_) <0.05. PASS 11.0 (NCSS, USA) was used to test the power analysis of sample size. The correlation coefficient (*r*) between TEs and OxS parameters was analyzed by Spearman correlation. When *P* < 0.0167 (adjusted *α* value by Bonferroni correction), the absolute value of the correlation coefficient ∣*r*_*s*_ | <0.2 indicates weak correlation; 0.2 ≤ ∣*r*_*s*_ | <0.4 indicates mild correlation; 0.4 ≤ ∣*r*_*s*_ | <0.6 indicates moderate correlation; 0.6 ≤ ∣*r*_*s*_ | <0.8 indicates strong correlation; ∣*r*_*s*_ | ≥0.8 indicates extremely strong correlation [32]. Multinomial logistic regression was used to analyze the risks of TEs and OxS parameters for different cancers, and the odds ratio (OR) was statistically significant with *P* < 0.007 (adjusted *α* value by Bonferroni correction when making a crude analysis) or *P* < 0.006 (adjusted *α* value by Bonferroni correction when making a reanalysis adjusted by age and sex).

## 3. Results

### 3.1. Power Analysis of Sample Size

The minimum sample size of 9 cancer subgroups was 107 cases, and the maximum was 156 cases. Take 10 cases as a step, to verify whether 178 healthy volunteers reach sufficient sample size in the range of 100~160. Take *r*1 = 0 (the null hypothesis), *r*2 = 0.5 (the expected correlation coefficient), and *α* = 0.05. The statistical results were power = 0.99154 ~ 0.99886, which indicated that the sample size of 178 cases in the control group was adequate.

### 3.2. Basic Information and Laboratory Results of the Subjects

The baseline data and observed indicators of the two groups were compared, and the results are shown in Table 1. There was no statistically significant difference only in the zinc level between these two groups (*z* = 0.472, *P* = 0.637), and there was statistically significant difference in other TEs and OxS parameters between these two groups (all *P* < 0.05). In the TEs, Cu (*z* = 8.647, *P* < 0.001) increased, Fe (*z* = −2.218, *P* = 0.027), and Se (*z* = −5.586, *P* < 0.001) decreased. Among OxS parameters, TOS (*z* = 10.667, *P* < 0.001) and OSI (*z* = 11.121, *P* < 0.001) increased, and TAS (*z* = −9.112, *P* < 0.001) decreased. The results show that OxS is common in cancer patients.

### 3.3. TE Levels in Patients with Different Types of Cancer

**Figure 1:**
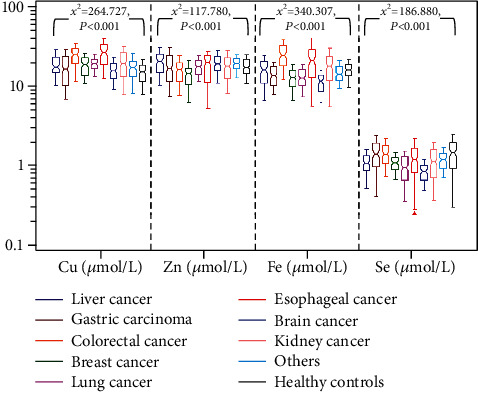
Notched Box-and-Whisker plot for the TE levels of all subjects. Note: the notch represents the median level, and the upper and lower lines of Box represent *P*_75_ and *P*_25_, respectively. Compared with healthy controls, cancer patients are generally accompanied by an increase in Cu and a decrease in Se. Zn decreases in patients with a variety of cancer, and the change of Fe varies with patients with different types of cancer.

According to the primary site of the tumor, the patients were divided into 9 subgroups (details are shown in Subjects). The comparison in TE levels between patient group and healthy control group was shown in Figure 1 and Table 2. Compared with healthy control group, patients in subgroups with liver cancer (*z* = 4.682, *P*_adj_ < 0.001), colorectal cancer (*z* = 11.473, *P*_adj_ < 0.001), breast cancer (*z* = 4.943, *P*_adj_ < 0.001), lung cancer (*z* = 6.762, *P*_adj_ < 0.001), esophageal cancer (*z* = 12.314, *P*_adj_ < 0.001), kidney cancer (*z* = 6.512, *P*_adj_ < 0.001), and other cancer (*z* = 3.441, *P*_adj_ = 0.026) had a significant increase in serum Cu levels. In addition, patients in subgroups with gastric carcinoma (*z* = 2.826, *P*_adj_ = 0.212) and brain cancer (*z* = 1.322, *P*_adj_ = 1.000) had no significant difference in serum Cu levels. Patients in breast cancer subgroup (*z* = −6.356, *P*_adj_ < 0.001) had a significant decrease in Zn level, and there was no significant change in other tumor subgroups (∣*z* | = 0.092 ~ 3.140, *P*_adj_ = 0.076 ~ 1.000). Patients in colorectal cancer (*z* = 7.792, *P*_adj_ < 0.001) and esophageal cancer (*z* = 3.410, *P*_adj_ = 0.029) subgroups had a significant increase in Fe levels, and patients in gastric carcinoma (*z* = −3.554, *P*_adj_ = 0.017), breast cancer (*z* = −5.657, *P*_adj_ < 0.001), lung cancer (*z* = −5.320, *P*_adj_ < 0.001), and brain cancer (*z* = −8.153, *P*_adj_ < 0.001) subgroups had a significant decrease in Fe levels. However, patients in liver cancer, kidney cancer, and other cancer subgroups (∣*z* | = 1.010 ~ 2.287, *P*_adj_ = 0.998 ~ 1.000) have no significant difference in Fe level. Patients in liver cancer (*z* = −4.468, *P*_adj_ < 0.001), breast cancer (*z* = −4.880, *P*_adj_ < 0.001), lung cancer (*z* = −7.312, *P*_adj_ < 0.001), brain cancer (*z* = −9.746, *P*_adj_ < 0.001), and kidney cancer (*z* = −3.996, *P*_adj_ = 0.003) subgroups had a significant decrease in Se level. In addition, patients in gastric carcinoma, colorectal cancer, esophageal cancer, and others subgroups (∣*z* | = 0.526 ~ 3.143, *P*_adj_ = 0.075 ~ 1.000) had no significant change in Se level. The results showed that the serum TE levels of cancer patients change.

When healthy subjects were excluded, the Kruskal-Wallis test statistics showed that the detected TEs, such as Cu (*χ*^2^ = 198.664, *P* < 0.001), Zn (*χ*^2^ = 112.810, *P* < 0.001), Fe (*χ*^2^ = 308.447, *P* < 0.001), and Se (*χ*^2^ = 167.064, *P* < 0.001), were statistically different among patients with different types of cancer. Among them, the Cu level was the highest in esophageal cancer, followed by colorectal cancer, kidney cancer, lung cancer, breast cancer, liver cancer, others, gastric carcinoma, and brain cancer. Zn level was the highest in liver cancer, followed by esophageal cancer, other cancer, brain cancer, kidney cancer, lung cancer, gastric carcinoma, colorectal cancer, and breast cancer. Fe level was the highest in colorectal cancer, followed by esophageal cancer, kidney cancer, liver cancer, other cancer, gastric carcinoma, breast cancer, lung cancer, and brain cancer. Se level was the highest in gastric carcinoma, followed by colorectal cancer, others, esophageal cancer, kidney cancer, breast cancer, liver cancer, lung cancer, and brain cancer. These results indicate that the changes of TEs are different among patients with different types of cancer.

### 3.4. OxS Parameters in Patients with Different Types of Cancer

**Figure 2:**
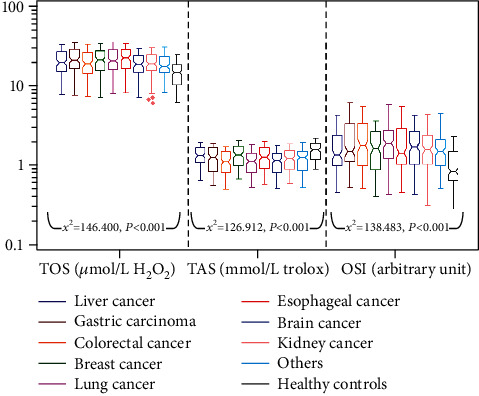
Notched Box-and-Whisker plot for OxS parameters of all subjects. Note: the notch represents the median level, and the upper and lower lines of Box represent *P*_75_ and *P*_25_, respectively. Compared with healthy controls, cancer patients show an increase in TOS and OSI and a decrease in TAS. This indicates that OxS is common in cancer patients.

The concentrations of OxS parameters in subjects were shown in Figure 2, and the comparison between 9 cancer subgroups and healthy control group was shown in Table 3. Compared with healthy control group, all 9 cancer subgroups had a significant increase in TOS (*z* = 4.976 ~ 9.245, all *P*_adj_ < 0.001) and OSI (*z* = 6.228 ~ 9.909, all *P*_adj_ < 0.001) and a significant decrease in TAS (*z* = −4.194 ~ −8.517, all *P*_adj_ < 0.001). These results indicated that OxS occurred in cancer patients.

When healthy subjects were excluded, Kruskal-Wallis test showed that there were statistical differences in TOS (*χ*^2^ = 35.862, *P* < 0.001) and TAS (*χ*^2^ = 45.593, *P* < 0.001) among patients with different types of tumors. However, there were no statistical differences in OSI (*χ*^2^ = 14.072, *P* = 0.080). Among them, the TOS level of other subgroup was lower than that of gastric carcinoma (*z* = −3.949, *P*_adj_ = 0.003), breast cancer (*z* = −3.415, *P*_adj_ = 0.023), lung cancer (*z* = −3.927, *P*_adj_ = 0.003), and esophageal cancer (*z* = −3.964, *P*_adj_ = 0.002) subgroups. There was no significant difference in TOS level among other subgroups (*z* = 0.005 ~ 2.980, all *P*_adj_ = 0.104 ~ 1.000). TAS level in breast cancer and liver cancer subgroups was higher than that in colorectal cancer (*z* = 4.601 and 3.715, *P*_adj_ < 0.01 and =0.007), lung cancer (*z* = 4.651 and 3.707, *P*_adj_ < 0.001 and =0.008), and brain cancer (*z* = 4.363 and 3.489, *P*_adj_ < 0.001 and =0.017) subgroups. There was no significant difference in TAS level among other subgroups (*z* = 0.003 ~ 3.148, all *P*_adj_ = 0.059 ~ 1.000). These results indicated that there was significant difference in OxS level among patients with different types of cancer.

### 3.5. Correlation between TEs and OxS Parameters

Spearman was used to analyze the correlation between TEs and OxS parameters in both patient group and the healthy control group (Table 4). In the patient group, Cu showed a moderate positive correlation with TOS (*r*_*s*_ = 0.508, *P* < 0.001) and OSI (*r*_*s*_ = 0.536, *P* < 0.001) and a moderate negative correlation with TAS (*r*_*s*_ = −0.449, *P* < 0.001). Zn showed a mild negative correlation with TOS (*r*_*s*_ = −0.274, *P* < 0.001) and OSI (*r*_*s*_ = −0.337, *P* < 0.001) and a mild positive correlation with TAS (*r*_*s*_ = 0.324, *P* < 0.001). Fe has a mild positive correlation with TOS (*r*_*s*_ = 0.229, *P* < 0.001) and OSI (*r*_*s*_ = 0.265, *P* < 0.001) and a mild negative correlation with TAS (*r*_*s*_ = −0.250, *P* < 0.001). Se showed a weak negative correlation with TOS (*r*_*s*_ = −0.159, *P* < 0.001) and OSI (*r*_*s*_ = −0.186, *P* < 0.001) and a weak positive correlation with TAS (*r*_*s*_ = 0.177, *P* < 0.001). In the healthy control group, Cu, Zn, Fe, and Se were not correlated with TOS, TAS, or OSI (all *P* > 0.0167). These results indicated that the relationship between TE levels and OxS in patients group is different from that in healthy control group.

Spearman statistical analysis was used to further analyze the correlation between TEs and OxS parameters of different cancer subgroups, and the results are shown in Table 5. Due to the complex composition of tumor types in the other subgroup, it is not described here. The relationships between Cu level and TOS, TAS, or OSI showed a moderate-to-strong correlation in liver cancer, gastric carcinoma, colorectal cancer, breast cancer, lung cancer, esophageal cancer, and brain cancer subgroups (∣*r*_*s*_ | = 0.497 ~ 0.795, all *P* < 0.001) and a mild correlation in kidney cancer subgroup (∣*r*_*s*_ | = 0.305 ~ 0.344, all *P* < 0.001). The relationships between Zn level and TOS, TAS, or OSI were not correlated in the brain cancer subgroup and showed the mild-to-moderate correlation in other cancer subgroups (∣*r*_*s*_ | = 0.231 ~ 0.521, all *P* < 0.0167). The relationship between Fe level and TOS, TAS, or OSI showed a moderate-to-strong correlation in lung cancer and esophageal cancer subgroups (∣*r*_*s*_ | = 0.529 ~ 0.760, all *P* < 0.001) and a mild-to-moderate correlation in liver cancer, colorectal cancer, and breast cancer subgroups (∣*r*_*s*_ | = 0.214 ~ 0.371, all *P* < 0.0167) and showed no correlation in gastric carcinoma, brain cancer, and kidney cancer subgroups. There was no correlation between Se level and TOS, TAS, or OSI in liver cancer, gastric carcinoma, colorectal cancer, breast cancer, and brain cancer subgroups and a weak-to-moderate correlation between them in lung cancer, esophageal cancer, and kidney cancer subgroups (∣*r*_*s*_ | = 0.192 ~ 0.408, all *P* < 0.0167). The results showed that the correlation between TE levels and OxS was different in patients with different types of cancer.

### 3.6. Multinomial Logistic Regression of Observed Biomarker in Patients with Different Types of Cancer

Multinomial logistic regression was used to analyze the risk of cancer. Firstly, 7 parameters of 4 TEs and 3 OxS parameters were adjusted, and *P* = 0.05/7 ≈ 0.007 was considered as statistical significance. After adding age and gender, a total of 9 participants were adjusted, and *P* = 0.05/9 ≈ 0.006 was considered as statistical significance. Taking cancer type as the dependent variable, healthy control as a reference population, age and sex as covariates, the cancer risks of 4 TEs and 3 OxS parameters were analyzed in nine cancer subgroups by multinomial logistic regression before and after age and sex adjustment. Odds ratios (ORs) were obtained before and after the adjustment of age and gender (Table 6).

After adjustment for age and sex, multinomial logistic regression showed that the decrease in Se (OR = 0.39, *P* < 0.001) and the increase in TOS (OR = 1.21, *P* = 0.006) increased the risk of liver cancer. The decrease in Fe (OR = 0.85, *P* < 0.001) and the increase in TOS (OR = 1.19, *P* = 0.001) and OSI (OR = 4.64, *P* < 0.001) increased the risk of gastric carcinoma. The decrease in Zn (OR = 0.83, *P* < 0.001) and the increase in Cu (OR = 1.29, *P* < 0.001) and Fe (OR = 1.18, *P* = 0.001) increased the risk of colorectal cancer. The decrease in Zn (OR = 0.81, *P* < 0.001) and Fe (OR = 0.83, *P* < 0.001) and the increase in TOS (OR = 1.34, *P* < 0.001) and OSI (OR = 2.56, *P* < 0.001) increased the risk of breast cancer. The decrease in Fe (OR = 0.80, *P* < 0.001) and Se (OR = 0.28, *P* < 0.001) and the increase in OSI (OR = 3.29, *P* < 0.001) increased the risk of lung cancer. The increase in Cu (OR = 1.44, *P* < 0.001) and TOS (OR = 2.82, *P* = 0.003) increased the risk of esophageal cancer. The decrease in Cu (OR = 0.73, *P* < 0.001), Fe (OR = 0.68, *P* < 0.001), Se (OR = 0.22, *P* < 0.001), and TAS (OR = 0.20, *P* < 0.001) and the increase in TOS (OR = 1.25, *P* = 0.003) and OSI (OR = 4.70, *P* < 0.001) increased the risk of brain cancer. The decrease in Zn (OR = 0.91, *P* = 0.001) and Se (OR = 0.45, *P* < 0.001) and the increase in Cu (OR = 1.10, *P* < 0.001) and OSI (OR = 4.14, *P* < 0.001) increased the risk of kidney cancer. The decrease in Fe (OR = 0.92, *P* = 0.002), Se (OR = 0.34, *P* < 0.001), and OSI (OR = 4.35, *P* < 0.001) increased the risk of other cancers. These experimental results showed that the levels of TEs and OxS in cancer patients are different, which causes the difference in risks of cancer occurrence.

## 4. Discussion

The results of this study show that OxS occurs in cancer patients, which may be the result of the consumption of antioxidants due to the antioxidants in the body. OxS is a physiological state that produces high levels of reactive oxygen species (ROS) and free radicals in the process of antioxidant metabolism [33]. OxS affects oncogene signaling pathway, which results in a large number of ROS production. It may use potential mutation and genomic variation of tumor cells to stimulate tumor progression [33]. It was previously reported that OxS is closely related to the occurrence and development of non-small-cell lung cancer. The cellular response of lung cancer patients to OxS is related to many metabolic pathways/genes of antioxidant enzymes, and these enzymes include superoxide dismutase, glutathione peroxidase, glucocorticoid receptor, heme oxygenase, and hypoxia inducible factor-1 genes *α* [34]. OxS is a key factor in the occurrence and development of colitis-associated colorectal cancer (CAC); ROS plays an important role in the trigger, promotion, and progression of CAC [35]. But a study of OxS in oropharyngeal cancer (OC) patients with or without periodontitis showed that salivary TAS levels were significantly higher in OC with periodontitis patients compared with healthy nonperiodontitis, healthy periodontitis, and nonperiodontitis with OC patients. Salivary TOS levels were significantly higher in OC with periodontitis patients compared with OC without periodontitis patients. But there was no significant difference in saliva OSI ratio among the subjects [36]. In tumor microenvironment, tumor cells show abnormal redox balance. ROS affect the development of tumor in an extremely contradictory way. An appropriate amount of ROS can promote tumorigenesis and support the transformation and proliferation of tumor cells, but high ROS can lead to the death of tumor cells [37].

The World Health Organization Human Nutrition Expert Committee announced in 1996 that there are 13 TEs necessary for human health and nutrition [38], including Cu (Copper), Zn (Zinc), Fe (Iron), Se (Selenium), and Iodine, Molybdenum, Chromium, Cobalt, Manganese, Silicon, Nickel, Boron, and Vanadium. Among them, the relationship between Cu, Zn, Fe, Se, and OxS has attracted more attention. For many years, many scholars wanted to explore the mechanism of cancer occurrence and development by analyzing the relationship between TEs and OxS [39, 40]. In this study, four TEs of Cu, Zn, Fe, and Se were measured. The changes in the four types of TEs in the body may be related to the occurrence and development of cancer. According to previous studies on single oxides (antioxidants) and/or their metabolites, these TEs can promote (or inhibit) occurrence and development of cancer by enhancing (or antagonizing) OxS [41].

Excess Cu may lead to cancer occurrence. Its carcinogenic mechanism may be related to copper zinc superoxide dismutase (Cu/Zn SOD) activating dioxygen and ceruloplasmin to scavenge free radicals by affecting the process of iron metabolism [42, 43]. Many types of cancer are characterized by increased intratumoral copper and/or altered systemic copper distribution. Copper is involved in tumorigenesis and development, including tumor growth, angiogenesis, and metastasis [44]. Zn and Se are not only good antioxidants, but also the active centers or essential components of many proteins and enzymes, such as maintaining the stability of their molecular structure and playing important physiological functions [45]. In addition, Zn can protect thiol and other chemical groups from oxidation [45]. Zn has a wide range of anticancer effects. Its anticancer defense mechanism is not only related to its antioxidant properties, but also affects the immune system, transcription factors, apoptosis, cell differentiation and proliferation, nucleic acid synthesis and repair, enzyme activation or inhibition, cell signal regulation, cell structure, and membrane stability [46]. Se is an important molecular composition of many antioxidant substances (enzymes), such as glutathione peroxidase, thioredoxin reductase, methionine sulfoxide reductase1, and endoplasmic reticulum-selenoproteins [47, 48]. These enzymes can enhance antioxidant activity by blocking lipid peroxidation, reducing organic peroxides, or destroying epoxides produced in the body. Therefore, Se can eliminate the excess free radicals generated by the free radical chain reaction triggered by oxidants in the body [49, 50]. It is reported that the negative correlation between selenium and prostate cancer risk may also reduce the risk of lung cancer [51]. However, this study also found that Se deficiency was associated with the risk of liver cancer, brain cancer, and kidney cancer, especially brain cancer. In this study, further studies will be conducted on the OxS response results (partially necessary changes in TEs and OxS parameters) produced by the subjects maintaining the oxidation-antioxidant balance in the body. As a result, Cu increases or has an increasing trend in all cancer patients, and serum Se decreases or has a decreasing trend. Zn increases in many cancer patients, and Zn decreases in individual patients. The inconsistent changes of Zn in different cancer patients may be due to different levels of antioxidant substance consumption.

The carcinogenic risk of Fe is heterogeneous. When Fe is overloaded, it can lead to the formation of FR, lipid peroxidation, DNA, and protein damage through the exchange between its different oxidation forms and promote the occurrence and development of tumors [52]. Second, Fe poisoning can lead to the death of cancer cells through membrane lipid peroxidation and inhibit the occurrence and development of tumors [53]. Third, because cancer cells are more iron-dependent than normal cells, cancer cells remodel the iron metabolism pathway to enhance Fe consumption during their growth and replication [52, 53]. Tumor patients may also have insufficient serum Fe. Fe is very unusual in relation to cancer. Fe overload is associated with increasing incidence rate and risk of cancer. Fe is involved in the initiation, growth, progression, and metastasis of cancer. However, both Fe excess and iron depletion can be utilized to antitumor therapy [54]. In patient group, levels of serum Fe in patients with liver cancer, lung cancer, brain cancer, breast cancer, and gastric cancer are lower than that in the control group, and Fe excess is inversely associated with cancer risk in breast, stomach, lung, and brain cancers. This can be used to prove that these cancer patients may consume a large amount of Fe storage in the body. The serum iron level of patients with colorectal cancer and esophageal cancer is significantly higher than that of the control group. This may be because that these patients are in the stage of tumor development such as invasion or metastasis.

We explored the relationship between these four types of essential TEs and OxS in cancer patients. Through correlation statistical analysis, this study found that the OxS occurrence is consistent with the increase of Cu and Fe levels and the decrease of Se levels in breast cancer, lung cancer, esophageal cancer, and other cancer subgroups. Compared with the case of healthy controls, there is no significant difference in these TE levels, i.e., Cu in gastric carcinoma and brain cancer patients, Fe in liver cancer patients, and Zn in liver cancer, gastric carcinoma, colorectal cancer, lung cancer, esophageal cancer, and kidney cancer patients. However, TEs showed a strong correlation with OxS parameters, indicating that these TEs are involved in the whole process of OxS. In addition, although Fe and Se significantly decrease in patients with gastric carcinoma and brain cancer, there were no correlation between them and OxS parameters. This indicates that the occurrence of OxS must involve other oxidizing or antioxidant substances. Therefore, the correlation between OxS parameters (including TOS, TAS, and OSI) in cancer patients and the four observed TEs (Cu, Zn, Fe, and Se) does not completely depend on the changes in the levels of these serum TEs in patients. The reason is that changes in TE only affect one or some metabolic processes of the oxidation-antioxidant system. In addition to the oxidation-reduction metabolites involved in TE for the overall oxidation state or antioxidant state of the body, the existence and synergistic effects of other oxidation or antioxidant substances need to be considered to accurately identify and judge whether the OxS occurs in body. Therefore, it is necessary to study the occurrence of OxS in subjects, the determination of TOS and TAS levels, and the calculation of OSI.

Many studies are to detect certain oxidative/antioxidant substances or their metabolic end products in patient samples to determine whether OxS occurs or not. However, the body's oxidation-antioxidant system is complex, and its oxidation (or antioxidant) capacity cannot be simply summarized by the level or capacity of one or several oxidizing (or antioxidizing) substances or their metabolites. At present, there are still many unrecognized oxidizing (or antioxidant) substances in the human body. Even if it is already known, it may be difficult or even impossible to detect. Moreover, not only can different oxidation (or antioxidant) substances exert the same oxidation (or antioxidant) effect, but also the same kind of oxidation (or antioxidant) can synergize with each other, exerting a stronger superimposing effect than simple weighting [55]. Therefore, due to different observed indicators, different researchers or research methods have inconsistent or even completely different results. Choosing the correct measurement parameters to evaluate the method of OxS is important to reach correct conclusions [56]. In this study, we measured the patient's serum TAS and TOS levels and calculated OSI. Analyzing the OxS status in the subjects from the overall level can comprehensively and truly reflect the body's oxidation and antioxidant levels. Compared with TAS and TOS, OSI can more intuitively reflect whether the subject's oxidation-antioxidant system is balanced. When the changes of oxidation and antioxidant substances are inconsistent, the body's oxidation-antioxidation balance is broken. This can cause OxS to occur and OSI to change. Therefore, the quantitative measurement of TAS and TOS and the calculation of the ratio OSI are important methods to accurately evaluate the OxS of the subject's body. Only in this way can we study the relationship between tumor occurrence, tumor development, and OxS, understand the true levels of oxidation and antioxidant substances in patients, and correctly evaluate OxS level.

Due to the large number of tumor types involved in this research, it is difficult to set up disease controls one by one for comparative analysis or long-term disease follow-up observation. Therefore, the causal relationship cannot be drawn. In the future, we will study a certain cancer by setting up healthy controls and related disease controls, grading, and staging tumor. Then, we can observe and analyze the characteristics of changes in the levels of TEs in the subject's body or take in-depth study of the pathological mechanism of OxS and TE changes by changing the levels of TEs to establish animal models.

In conclusion, a correlation was observed between OxS and TEs in blood of cancer patients. This means that the occurrence of OxS in cancer is accompanied by the change of some TEs. Different types of tumors have different types and degrees of changes in TEs. However, the relationship between the changes of TEs and the occurrence of OxS in cancer patients needs further study.

## Figures and Tables

**Table 1:** Basic demographical data and laboratory results of all subjects.

	Healthy control (*n* = 178)	Cancer (*n* = 1143)	*χ* ^2^/*z*/*t*	*P*
Sex (male/female)	95/83	679/464	2.069	0.150
Age (years)	50.3 ± 14.7	52.4 ± 13.5	1.835	0.067
Type of work			8.363	0.137
Government staff	13 (7.3)	57 (5.0)		
Enterprise manager	11 (6.2)	115 (10.1)		
Mental worker	48 (27.0)	377 (33.0)		
Manual worker	39 (21.9)	203 (17.8)		
Peasantry	25 (14.0)	166 (14.5)		
Others	42 (23.6)	225 (19.7)		
Academic career			5.351	0.148
Junior school or below	22 (12.4)	190 (16.6)		
High school or technical secondary school	74 (41.6)	404 (35.3)		
Junior college	63 (35.4)	381 (33.3)		
Bachelor degree or above	19 (10.7)	168 (14.7)		
Hobby				
Drinker^∗^	45 (25.3)	328 (28.7)	2.443	0.118
Smoker^∗∗^	67 (37.6)	521 (45.6)	3.618	0.057
TE levels				
Cu (*μ*mol/L)	14.98 (11.28, 18.20)	18.59 (14.32, 23.45)	8.647	<0.001
Zn (*μ*mol/L)	17.09 (14.38, 21.59)	17.37 (13.18, 21.74)	0.472	0.637
Fe (*μ*mol/L)	15.70 (13.34, 18.59)	14.72 (10.96, 18.87)	-2.218	0.027
Se (*μ*mol/L)	1.47 (0.94, 1.98)	1.12 (0.83, 1.44)	-5.586	<0.001
OxS parameters				
TOS (*μ*mol/L H_2_O_2_)	14.56 (10.28, 18.02)	19.58 (15.11, 26.43)	10.667	<0.001
TAS (mmol/L Trolox)	1.59 (1.19, 1.92)	1.26 (0.91, 1.57)	-9.112	<0.001
OSI (arbitrary unit)	0.85 (0.65, 1.49)	1.59 (1.03, 2.68)	11.121	<0.001

Note: *t*-test is used for age, Mann-Whitney test is used for TEs and OxS parameters, and Chi-squared test is used for other observed indexes. ^∗^A little, each time does not exceed 20 ml, not exceeding 50 ml/day, and above 50 degrees of spirit. ^∗∗^Occasionally, no more than 2 cigarettes in 4 hours and no more than 5 cigarettes/day.

**Table 2:** Serum levels of four trace elements in patients with different types of cancer and healthy controls.

Subjects	*n*	Cu (*μ*mol/L)	Zn (*μ*mol/L)	Fe (*μ*mol/L)	Se (*μ*mol/L)
Liver cancer	107	17.14^∗^	20.23	15.74	1.09^∗^
Gastric carcinoma	119	16.13	16.63	13.49^∗^	1.42
Colorectal cancer	120	24.56^∗^	15.91	24.28^∗^	1.41
Breast cancer	128	18.36^∗^	14.23^∗^	12.74^∗^	1.10^∗^
Lung cancer	150	18.89^∗^	17.35	12.71^∗^	0.96^∗^
Esophageal cancer	117	26.08^∗^	19.75	20.66^∗^	1.21
Brain cancer	115	15.40	18.75	11.41^∗^	0.87^∗^
Kidney cancer	156	18.92^∗^	17.59	17.51^∗^	1.14^∗^
Others	131	16.85^∗^	19.00	13.97	1.21
Healthy control	178	14.98	17.09	15.69	1.47

*χ* ^2^, *P*		264.727, <0.001	117.780, <0.001	340.307, <0.001	186.880, <0.001

(*χ*^2^, *P*)		198.664, <0.001	112.810, <0.001	308.447, <0.001	167.064, <0.001

Note: Kruskal-Wallis test is used for comparison between groups. Mean rank multiple comparison of Kruskal-Wallis test is used for pairwise comparison. “*χ*^2^, *P*” row: all subjects (including healthy subjects), Kruskal-Wallis test statistical analysis result. “(*χ*^2^, *P*)” row: excluding healthy subjects, Kruskal-Wallis test statistical analysis results between patients with different types of cancers. ^∗^Compared with the case of healthy control group, there is a statistical significant difference (*P*_adj_ < 0.05).

**Table 3:** Serum OxS parameter levels in patients with different types of cancer and healthy controls.

Subjects	*n*	TOS (*μ*mol/L H_2_O_2_)	TAS (mmol/L Trolox)	OSI (arbitrary unit)
Liver cancer	107	19.42^∗^	1.34^∗^	1.38^∗^
Gastric carcinoma	119	20.89^∗^	1.28^∗^	1.51^∗^
Colorectal cancer	120	18.78^∗^	1.12^∗^	1.80^∗^
Breast cancer	128	21.10^∗^	1.38^∗^	1.64^∗^
Lung cancer	150	20.42^∗^	1.14^∗^	1.90^∗^
Esophageal cancer	117	22.24^∗^	1.29^∗^	1.46^∗^
Brain cancer	115	18.45^∗^	1.17^∗^	1.72^∗^
Kidney cancer	156	18.78^∗^	1.23^∗^	1.60^∗^
Others	131	17.33^∗^	1.27^∗^	1.51^∗^
Healthy control	178	14.56	1.59	0.85

*χ* ^2^, *P*		146.400, <0.001	126.912, <0.001	138.483, <0.001

(*χ*^2^, *P*)		35.862, <0.001	45.593, <0.001	14.072, 0.080

Note: Kruskal-Wallis test is used for comparison between groups. Mean rank multiple comparison of Kruskal-Wallis test is used for pairwise comparison. “*χ*^2^, *P*” row: all subjects (including healthy subjects), Kruskal-Wallis test statistical analysis result. “(*χ*^2^, *P*)” row: excluding healthy subjects, Kruskal-Wallis test statistical analysis results between patients with different types of cancers and Kruskal-Wallis test statistical analysis results after excluding healthy subjects. ^∗^Compared with the case of healthy control group, there is a statistical significant difference (*P*_adj_ < 0.001).

**Table 4:** Correlation between each TE and OxS parameter in serum of all subjects.

	TOS		TAS		OSI	
	*r*_*s*_	*P*	*r*_*s*_	*P*	*r*_*s*_	*P*
Cancer patients (*n* = 1143)						
Cu	0.508	<0.001	-0.449	<0.001	0.536	<0.001
Zn	-0.274	<0.001	0.324	<0.001	-0.337	<0.001
Fe	0.229	<0.001	-0.250	<0.001	0.265	<0.001
Se	-0.159	<0.001	0.177	<0.001	-0.186	<0.001
Healthy control (*n* = 178)						
Cu	0.117	0.121	-0.043	0.567	0.096	0.201
Zn	0.072	0.342	0.013	0.859	0.040	0.601
Fe	0.093	0.220	0.169	0.024	0.129	0.087
Se	0.123	0.102	-0.045	0.551	0.082	0.274

Note: Spearman correlation analysis is used. All TEs were correlated to OxS parameters (TOS, TAS, and OSI) in the patients group, whereas not in the healthy control group.

**Table 5:** Correlation between TEs and OxS parameter in different cancer subgroups.

	TOS		TAS		OSI	
	*r*_*s*_(95% CI)	*P*	*r*_*s*_(95% CI)	*P*	*r*_*s*_(95% CI)	*P*
Liver cancer (*n* = 107)						
Cu	0.689(0.574, 0.777)	<0.001	-0.739(-0.814. -0.638)	<0.001	0.795(0.713, 0.856)	<0.001
Zn	-0.382(-0.533, -0.207)	<0.001	0.491(0.332, 0.623)	<0.001	-0.458(-0.596, -0.293)	<0.001
Fe	0.270(0.085, 0.437)	0.005	-0.252(-0.422, -0.066)	0.009	0.306(0.123, 0.469)	0.001
Se	0.068(-0.124, 0.254)	0.489	0.138(-0.053, 0.319)	0.157	0.098(-0.094, 0.283)	0.315
Gastric carcinoma (*n* = 119)						
Cu	0.619(0.494, 0.719)	<0.001	-0.645(-0.739, -0.526)	<0.001	0.687(0.578, 0.771)	<0.001
Zn	-0.271(-0.430, -0.096)	0.003	0.266(0.091, 0.426)	0.003	-0.314(-0.468, -0.142)	<0.001
Fe	0.043(-0.138, 0.222)	0.640	-0.175(-0.345, 0.005)	0.056	0.099(-0.083, 0.274)	0.286
Se	-0.069(-0.246, 0.113)	0.459	0.117(-0.064, 0.291)	0.205	0.048(-0.133, 0.226)	0.602
Colorectal cancer (*n* = 120)						
Cu	0.636(0.515, 0.732)	<0.001	-0.520(-0.640, -0.376)	<0.001	0.632(0.510, 0.728)	<0.001
Zn	-0.316(-0.468, -0.145)	<0.001	0.340(0.171, 0.489)	<0.001	-0.362(-0.508, -0.195)	<0.001
Fe	0.329(0.159, 0.480)	<0.001	-0.347(-0.495, -0.179)	<0.001	0.371(0.205, 0.516)	<0.001
Se	0.013(-0.167, 0.191)	0.892	-0.046(-0.223, 0.135)	0.621	0.041(-0.139, 0.219)	0.655
Breast cancer (*n* = 128)						
Cu	0.580(0.452, 0.685)	<0.001	-0.534(-0.648, -0.398)	<0.001	0.627(0.509, 0.722)	<0.001
Zn	-0.259(-0.414, -0.090)	0.003	0.302(0.136, 0.452)	<0.001	-0.353(-0.496, -0.191)	<0.001
Fe	0.239(0.068, 0.396)	0.007	-0.214(-0.374, -0.042)	0.015	0.261(0.092, 0.416)	0.003
Se	-0.202(-0.363, -0.030)	0.022	0.203(0.030, 0.363)	0.022	-0.218(-0.377, -0.046)	0.014
Lung cancer (*n* = 150)						
Cu	0.583(0.467, 0.680)	<0.001	-0.519(-0.627, -0.391)	<0.001	0.631(0.523, 0.718)	<0.001
Zn	-0.278(-0.420, -0.124)	<0.001	0.339(0.189, 0.474)	<0.001	-0.342(-0.476, -0.192)	<0.001
Fe	0.674(0.576, 0.753)	<0.001	-0.685(-0.762, -0.590)	<0.001	0.760(0.683, 0.820)	<0.001
Se	-0.261(-0.405, -0.106)	0.001	0.219(0.061, 0.366)	0.007	-0.248(-0.393, -0.091)	0.002
Esophageal cancer (*n* = 117)						
Cu	0.657(0.540, 0.749)	<0.001	-0.647(-0.741, -0.527)	<0.001	0.714(0.612, 0.793)	<0.001
Zn	-0.498(-0.623, -0.348)	<0.001	0.491(0.340, 0.618)	<0.001	-0.521(-0.621, -0.396)	<0.001
Fe	0.529(0.385, 0.648)	<0.001	-0.585(-0.693, -0.451)	<0.001	0.644(0.523, 0.739)	<0.001
Se	-0.327(-0.480, -0.154)	<0.001	0.381(0.214, 0.526)	<0.001	-0.408(-0.549, -0.245)	<0.001
Brain cancer (*n* = 115)						
Cu	0.479(0.325, 0.609)	<0.001	-0.584(-0.693, -0.448)	<0.001	0.627(0.502, 0.727)	<0.001
Zn	0.145(-0.040, 0.319)	0.123	-0.147(-0.311, 0.025)	0.094	0.182(-0.001, 0.354)	0.051
Fe	0.107(-0.078, 0.284)	0.257	-0.075(-0.254, 0.110)	0.428	0.125(-0.060, 0.301)	0.183
Se	0.111(-0.047, 0.264)	0.167	-0.067(-0.248, 0.117)	0.473	0.142(-0.042, 0.317)	0.130
Kidney cancer (*n* = 156)						
Cu	0.342(0.195, 0.474)	<0.001	-0.305(-0.441, -0.155)	<0.001	0.344(0.198, 0.476)	<0.001
Zn	-0.231(-0.374, -0.077)	0.004	0.298(0.148, 0.435)	<0.001	-0.280(-0.419, -0.129)	<0.001
Fe	0.073(-0.085, 0.228)	0.365	-0.098(-0.251, 0.060)	0.224	0.096(-0.063, 0.249)	0.236
Se	-0.187(-0.358, -0.004)	0.045	0.234(0.080, 0.377)	0.003	-0.192(-0.339, -0.036)	0.016
Others (*n* = 131)						
Cu	0.365(0.206, 0.505)	<0.001	-0.297(-0.446, -0.132)	<0.001	0.397(0.242, 0.532)	<0.001
Zn	-0.364(-0.504, -0.206)	<0.001	0.200(0.017, 0.369)	0.032	-0.290(-0.440, -0.125)	<0.001
Fe	0.270(0.103, 0.422)	0.002	-0.246(-0.401, -0.078)	0.005	0.298(0.133, 0.447)	<0.001
Se	-0.378(-0.516, -0.221)	<0.001	0.279(0.112, 0.430)	0.001	-0.406(-0.540, -0.252)	<0.001

Note: Spearman correlation analysis is used.

**Table 6:** The multinomial regression of TEs and OxS parameters in different cancers.

Cancer	Index	Crude			Adjusted^∗^		
		OR(95% CI)	Wald *χ*^2^	*P*	OR(95% CI)	Wald *χ*^2^	*P*
Liver cancer	Cu	1.06(0.99, 1.12)	3.413	0.065	1.01(0.93, 1.10)	0.077	0.781
	Zn	1.03(0.98, 1.09)	1.380	0.240	1.07(0.98, 1.16)	2.430	0.119
	Fe	0.96(0.91, 1.01)	2.511	0.113	0.94(0.86, 1.02)	2.016	0.156
	Se	0.35(0.18, 0.57)	18.940	<0.001	0.39(0.17, 0.66)	13.361	<0.001
	TOS	1.22(1.11, 1.35)	16.758	<0.001	1.21(1.06, 1.38)	7.648	0.006
	TAS	0.47(0.11, 2.02)	1.027	0.311	0.40(0.05, 3.50)	0.684	0.408
	OSI	3.48(2.27, 5.35)	32.566	<0.001	1.54(0.89, 2.66)	2.396	0.122

Gastric carcinoma	Cu	0.95(0.89, 1.01)	3.195	0.074	0.94(0.88, 0.99)	4.025	0.045
	Zn	0.93(0.88, 0.98)	7.391	0.007	0.94(0.89, 1.01)	3.658	0.056
	Fe	0.86(0.81, 0.91)	25.315	<0.001	0.85(0.80, 0.92)	19.495	<0.001
	Se	1.11(0.66, 1.86)	0.147	0.701	1.28(0.74, 2.21)	0.754	0.385
	TOS	1.18(1.08, 1.29)	12.820	<0.001	1.19(1.07, 1.32)	10.179	0.001
	TAS	2.29(0.62, 8.54)	1.530	0.216	1.95(0.42, 9.03)	0.732	0.392
	OSI	7.45(4.92, 11.30)	89.644	<0.001	4.64(2.93, 7.32)	43.207	<0.001

Colorectal cancer	Cu	1.24(1.17, 1.32)	51.582	<0.001	1.29(1.15, 1.44)	20.029	<0.001
	Zn	0.80(0.76, 0.85)	49.473	<0.001	0.83(0.75, 0.92)	12.230	<0.001
	Fe	1.21(1.15, 1.27)	56.182	<0.001	1.18(1.07, 1.31)	10.547	0.001
	Se	0.64(0.35, 1.17)	2.078	0.149	1.06(0.36, 3.13)	0.010	0.921
	TOS	0.99(0.89, 1.10)	0.028	0.866	0.92(0.78, 1.09)	0.961	0.327
	TAS	0.35(0.08, 1.51)	1.980	0.159	0.40(0.03, 6.22)	0.425	0.515
	OSI	2.67(1.72, 4.15)	19.160	<0.001	1.04(0.66, 1.75)	0.305	0.581

Breast cancer	Cu	1.05(0.99, 1.11)	2.473	0.116	0.95(0.85, 1.05)	1.181	0.277
	Zn	0.79(0.74, 0.84)	61.402	<0.001	0.81(0.74, 0.89)	19.871	<0.001
	Fe	0.84(0.79, 0.89)	29.760	<0.001	0.83(0.75, 0.91)	13.515	<0.001
	Se	0.39(0.20, 0.55)	18.845	<0.001	0.53(0.22, 0.89)	4.753	0.029
	TOS	1.32(1.20, 1.46)	32.488	<0.001	1.34(1.17, 1.54)	17.573	<0.001
	TAS	0.52(0.12, 2.28)	0.742	0.389	0.22(0.02, 2.41)	1.547	0.214
	OSI	5.70(3.72, 8.72)	64.053	<0.001	2.56(1.48, 4.42)	11.377	<0.001

Lung cancer	Cu	1.05(0.99, 1.11)	2.450	0.117	0.95(0.86, 1.05)	1.092	0.296
	Zn	0.93(0.88, 0.98)	7.528	0.006	0.97(0.89, 1.06)	0.332	0.565
	Fe	0.82(0.77, 0.87)	41.438	<0.001	0.80(0.72, 0.88)	19.186	<0.001
	Se	0.26(0.13, 0.42)	45.183	<0.001	0.28(0.12, 0.44)	25.849	<0.001
	TOS	1.18(1.07, 1.29)	12.196	<0.001	1.19(1.04, 1.37)	6.717	0.010
	TAS	0.32(0.13, 0.55)	9.034	0.003	0.25(0.10, 0.49)	6.550	0.010
	OSI	7.08(4.67, 10.72)	85.428	<0.001	3.29(1.94, 5.58)	19.408	<0.001

Esophageal cancer	Cu	1.37(1.29, 1.46)	100.311	<0.001	1.44(1.29, 1.61)	41.301	<0.001
	Zn	0.89(0.85, 0.95)	14.667	<0.001	0.97(0.88, 1.06)	0.507	0.477
	Fe	1.09(1.04, 1.15)	12.382	<0.001	1.08(0.98, 1.19)	2.238	0.135
	Se	0.41(0.21, 0.59)	15.375	<0.001	0.46(0.19, 0.76)	6.115	0.013
	TOS	2.75(1.23, 5.54)	9.772	0.002	2.82(1.45, 6.49)	8.747	0.003
	TAS	1.11(1.01, 1.22)	4.350	0.037	0.97(0.83, 1.14)	0.131	0.718
	OSI	2.27(1.46, 3.54)	13.185	<0.001	1.06(0.76, 1.63)	1.705	0.192

Brain cancer	Cu	0.89(0.83, 0.95)	10.995	0.001	0.73(0.65, 0.82)	27.324	<0.001
	Zn	1.02(0.96, 1.08)	0.563	0.453	1.07(0.97, 1.18)	1.937	0.164
	Fe	0.71(0.65, 0.76)	73.692	<0.001	0.68(0.60, 0.77)	39.332	<0.001
	Se	0.22(0.11, 0.34)	60.802	<0.001	0.22(0.10, 0.36)	42.254	<0.001
	TOS	1.17(1.05, 1.30)	8.227	0.004	1.25(1.08, 1.44)	8.680	0.003
	TAS	0.22(0.10, 0.37)	17.296	<0.001	0.20(0.13, 0.32)	21.599	<0.001
	OSI	8.70(5.61, 13.50)	93.129	<0.001	4.70(2.70, 8.19)	29.917	<0.001

Kidney cancer	Cu	1.11(1.05, 1.17)	15.409	<0.001	1.10(1.05, 1.16)	15.717	<0.001
	Zn	0.92(0.88, 0.97)	11.220	0.001	0.91(0.87, 0.96)	11.094	0.001
	Fe	1.04(1.00, 1.09)	3.283	0.070	1.04(0.99, 1.09)	2.001	0.157
	Se	0.40(0.22, 0.54)	27.386	<0.001	0.45(0.25, 0.62)	28.107	<0.001
	TOS	1.08(0.99, 1.18)	2.871	0.090	1.06(0.97, 1.17)	1.666	0.197
	TAS	0.34(0.14, 0.59)	9.647	0.002	0.46(0.17, 0.72)	4.360	0.037
	OSI	3.59(2.39, 5.40)	37.702	<0.001	4.14(2.71, 6.32)	43.331	<0.001

Others	Cu	0.99(0.94, 1.05)	0.069	0.792	1.02(0.97, 1.07)	0.430	0.512
	Zn	1.01(0.96, 1.06)	0.158	0.691	1.00(0.95, 1.05)	0.016	0.899
	Fe	0.91(0.87, 0.96)	12.013	0.001	0.92(0.87, 0.97)	9.468	0.002
	Se	0.29(0.17, 0.50)	20.404	<0.001	0.34(0.20, 0.56)	17.572	<0.001
	TOS	1.06(0.97, 1.16)	1.488	0.223	1.03(0.94, 1.14)	0.403	0.526
	TAS	0.32(0.13, 0.60)	11.489	0.001	0.41(0.16, 0.74)	5.923	0.015
	OSI	4.61(3.04, 6.98)	52.138	<0.001	4.35(2.85, 6.65)	46.311	<0.001

Note: ^∗^Adjusted by age and sex.

## Data Availability

All data, models, and code generated or used during the study appear in the submitted article.
